# The tri-peptide GHK-Cu complex ameliorates lipopolysaccharide-induced acute lung injury in mice

**DOI:** 10.18632/oncotarget.11168

**Published:** 2016-08-10

**Authors:** Jeong-Ran Park, Hanbyeol Lee, Seok-In Kim, Se-Ran Yang

**Affiliations:** ^1^ Department of Thoracic and Cardiovascular Surgery, School of Medicine, Kangwon National University, Chuncheon, Republic of Korea; ^2^ Institute of Medical Science, Kangwon National University, Chuncheon, Republic of Korea; ^3^ Bioceltran Co., Ltd., Chuncheon, Republic of Korea

**Keywords:** GHK-Cu, acute lung, injury, inflammation, NF-κB p65, p38 MAPK

## Abstract

The tripeptide-copper complex glycyl-l-histidyl-l-lysine-Cu (II) (GHK-Cu) is involved in wound healing and tissue remodeling. Although GHK-Cu exhibits anti-aging and tissue renewing properties, its roles in acute lung injury (ALI)/acute respiratory distress syndrome (ARDS) are still unknown. Therefore, we examined the effects of GHK-Cu in lipopolysaccharide (LPS)-induced RAW 264.7 macrophages *in vitro* and ALI in mice *in vivo*. GHK-Cu treatment reduced reactive oxygen species (ROS) production, increased superoxide dismutase (SOD) activity while decreased TNF-α and IL-6 production through the suppression of NF-κB p65 and p38 MAPK signaling *in vitro* and *in vivo* model of ALI. Moreover, GHK-Cu attenuated LPS-induced lung histological alterations, suppressed the infiltration of inflammatory cells into the lung parenchyma in LPS-induced ALI in mice. Taken together, these findings demonstrate that GHK-Cu possesses a protective effect in LPS-induced ALI by inhibiting excessive inflammatory responses; accordingly it may represent a novel therapeutic approach for ALI/ARDS.

## INTRODUCTION

Acute lung injury (ALI), along with its most severe form, acute respiratory distress syndrome (ARDS), is a disorder of acute inflammation and tissue injury that is characterized by the loss of alveolar-capillary membrane integrity, excessive transepithelial neutrophil migration, and the release of pro-inflammatory and cytotoxic mediators [1, 2]. The incidence of ALI/ARDS ranges from 1.5 to 100 per 100,000 person-years, depending on various factors, including methodology, geography, definition, age, and associated clinical disorders [3]. It has been reported that long after lung injury, survivors have a significantly lower quality of life due to the burden of the injury and sequelae with pulmonary dysfunction [4]. ALI is mainly caused by direct lung injuries, such as the inhalation of toxic substances, and indirect systemic diseases, including sepsis, bacterial pneumonia, and severe trauma [5]. Despite advances in supportive care for survivors and improvements in anti-inflammatory interventions and treatments that inhibit the inflammatory process in lungs, the morbidity and mortality of ALI remains higher than acceptable [6].

Lipopolysaccharide (LPS, also termed endotoxin) is the major component of the outer membrane of gram-negative bacteria. It is recognized by the immune system as a marker for bacterial pathogen invasion and is responsible for the development of inflammatory responses. LPS could activate airway epithelial cells, neutrophils, and alveolar macrophages, resulting in the release of numerous inflammatory mediators, such as reactive oxygen species (ROS), tumor necrosis factor-α (TNF-α), and interleukin-6 (IL-6).

Among existing signaling pathways, the mitogen-activated protein kinase (MAPK) and nuclear factor-κB (NF-κB) signaling pathways are key regulators of inflammatory processes. Several studies have shown that NF-κB-dependent gene expression plays an important role in inflammatory responses and increases the expression of genes encoding cytokines [7, 8]. The mitogen-activated protein kinases (MAPKs) family, including extracellular signal-regulated kinase 1/2 (ERK1/2), p38 MAPK, and c-Jun NH2-terminal kinase (JNK1/2), has been shown to play a significant role in the mediation of signals triggered by cytokines, growth factors, and environmental stress and is involved in various cellular functions [9, 10]. In particular, p38 MAPK is activated by various pro-inflammatory and stress stimuli and thus plays an essential role in cellular responses, including inflammation, cell proliferation, and apoptosis. Furthermore, the role of p38 MAPK in such diseases has been examined, and its inhibitors are potential therapeutic agents for their treatment [11, 12].

Numerous studies have reported that inflammation may directly and indirectly affect lung endothelia and pulmonary hemodynamics by activating inflammatory cells, such as neutrophils, macrophages, and lymphocytes, and proinflammatory mediators [13]. Macrophages are generally an important component in immune defense. A role of macrophages in initiating and maintaining pulmonary inflammation in lung infection or injury has been convincingly demonstrated. Recent reports suggest that lung macrophages are important orchestrators of the termination and resolution of inflammation and initiate parenchymal repair processes that are essential for a return to homeostasis with normal gas exchange [14, 15]. Furthermore, it has been hypothesized that inhibiting IL-6 and TNF-α production in macrophages can serve as the basis for the development of anti-inflammatory drugs.

Oxidative damage due to inflammatory responses is a major cause of lung injury during ALI [16]. Furthermore, proinflammatory mediators, such as ROS, NO, and Cox-2, play a key role in the pathogenesis of many acute inflammatory diseases [17]. These responses may enable the identification of molecules that modulate lung inflammation in ALI/ARDS.

The human tripeptide Glycyl-l-histidyl-l-lysine (GHK) is present in the plasma, saliva, and urine and is used for wound treatment and skin care [18]. It is naturally occurring, nontoxic, and readily forms complexes with copper, regulating its metabolism and improving its bioavailability. GHK tripeptide and its copper (II)-chelated form (GHK-Cu) accelerate the process of regeneration, wound healing, and antioxidant and anti-inflammatory actions [19, 20]. GHK-Cu suppresses inflammation by lowering the level of acute-phase inflammatory cytokines, including TGF-β and TNF-α, and reduces oxidative damage by modulating iron levels [21, 22]. Recent studies have demonstrated an antioxidant and anti-inflammatory role of GHK-Cu to ameliorate skin damage; however, the effects of GHK-Cu on LPS-induced ALI are unknown. Therefore, we investigated whether GHK-Cu administration can attenuate lung injury via the suppression of inflammatory responses in a model of LPS-induced ALI. Furthermore, we determined the mechanism underlying the beneficial effects, including the roles of the NF-κB and MAPK pathways and oxidative enzymes.

## RESULTS

### GHK-Cu decreased ROS production and improved SOD activity in LPS-induced RAW 264.7 cells activation

**Figure 1:**
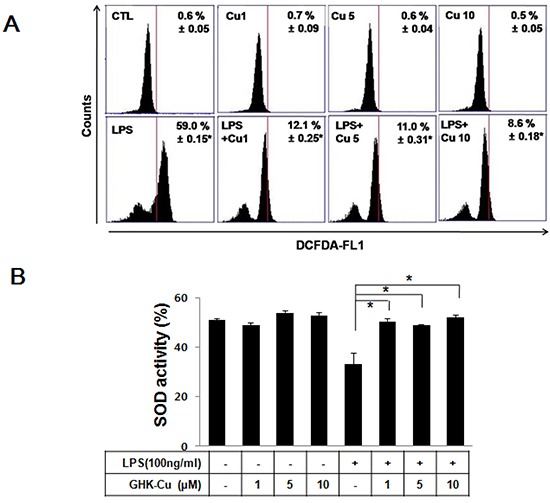
GHK-Cu decreased ROS production and increased SOD activity in LPS-induced RAW 264.7 cells activation. Four hours after LPS treatment with or without GHK-Cu pretreatment for 18 h, the cells were harvested and assayed **A.** The x-axis represent the intensity of intracellular DCFDA fluorescence, and the *y*-axis indicate the mean number of cells. **B.** Total intracellular SOD activity levels were detected using a colorimetric method. The results are representative of triplicate data. The values are presented as the means ± SD (n = 3). *p < 0.05, statistically significant difference. CTL, control.

According to a previous study, GHK-Cu can increase the level of antioxidant enzymes and SOD activity, supposedly by supplying copper, which is necessary for its function [23]. Macrophages play an important role in inflammatory events and promote the transcription of pro-inflammatory mediators. Therefore, we examined the effects of GHK-Cu on LPS-induced ROS production and SOD activity in RAW 264.7 cells. Cellular ROS was measured using DCFDA and flow cytometry. RAW 264.7 cells were treated with LPS with or without pretreatment with various concentrations of GHK-Cu. GHK-Cu did not affect cell proliferation any of the concertation examined (Supplementary Figure S1A). As shown in Figure 1A, the fluorescein derivative DCF was oxidized 59% more in LPS-treated cells compared to the control. However, pretreatment with GHK-Cu at 1, 5, and 10 μM significantly decreased ROS production. SOD is an antioxidant enzyme and is markedly decreased in LPS-induced ALI [24]. We determined SOD activity after treatment with LPS with or without pretreatment with various concentrations of GHK-Cu. SOD activity was significantly decreased by approximately 19 % in the LPS-treated cells compared to the control (Figure 1B). In contrast, SOD activity in cells pretreated with GHK-Cu (1, 5, and 10 μM) improved to nearly control levels. We also determined NO secretion, is known as regulator of inflammatory response, after LPS treatment with or without GHK-Cu pretreatment. In the present work, GHK-Cu did not change NO secretion (Supplementary Figure S1B).

### GHK-Cu attenuated the release of pro-inflammatory cytokines in LPS-induced RAW 264.7 cells activation

**Figure 2:**
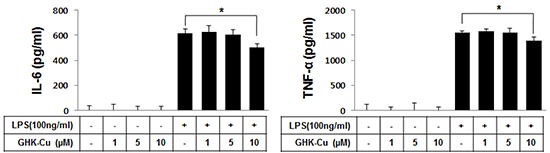
GHK-Cu downregulated the release of TNF-α and IL-6 in LPS-induced RAW 264.7 cells activation TNF-α and IL-6 levels in culture supernatants were determined by ELISA. These data showed that cells that were pretreated with 10 μM GHK-Cu exhibited significantly decreased secretion of TNF-α and IL-6. The data represent the means ± SD (n = 3). *p < 0.05, statistically significant difference. CTL, control.

To determine whether GHK-Cu reduces the release of inflammatory cytokines, the levels of TNF-α and IL-6 were confirmed by ELISA. Exposure of RAW 264.7 cells to LPS for 4 h significantly increased IL-6 and TNF-α secretion in the culture medium to 613.2 ± 35.1 and 1556.3 ± 23.3 pg/ml, respectively. The cells that were pretreated with 10 μM GHK-Cu significantly decreased secretion of these molecules (Figure 2).

### GHK-Cu blocked the LPS-induced nuclear translocation of NF-κB p65 and phosphorylation of NF-κB p65 in LPS-induced RAW 264.7 cells activation

**Figure 3:**
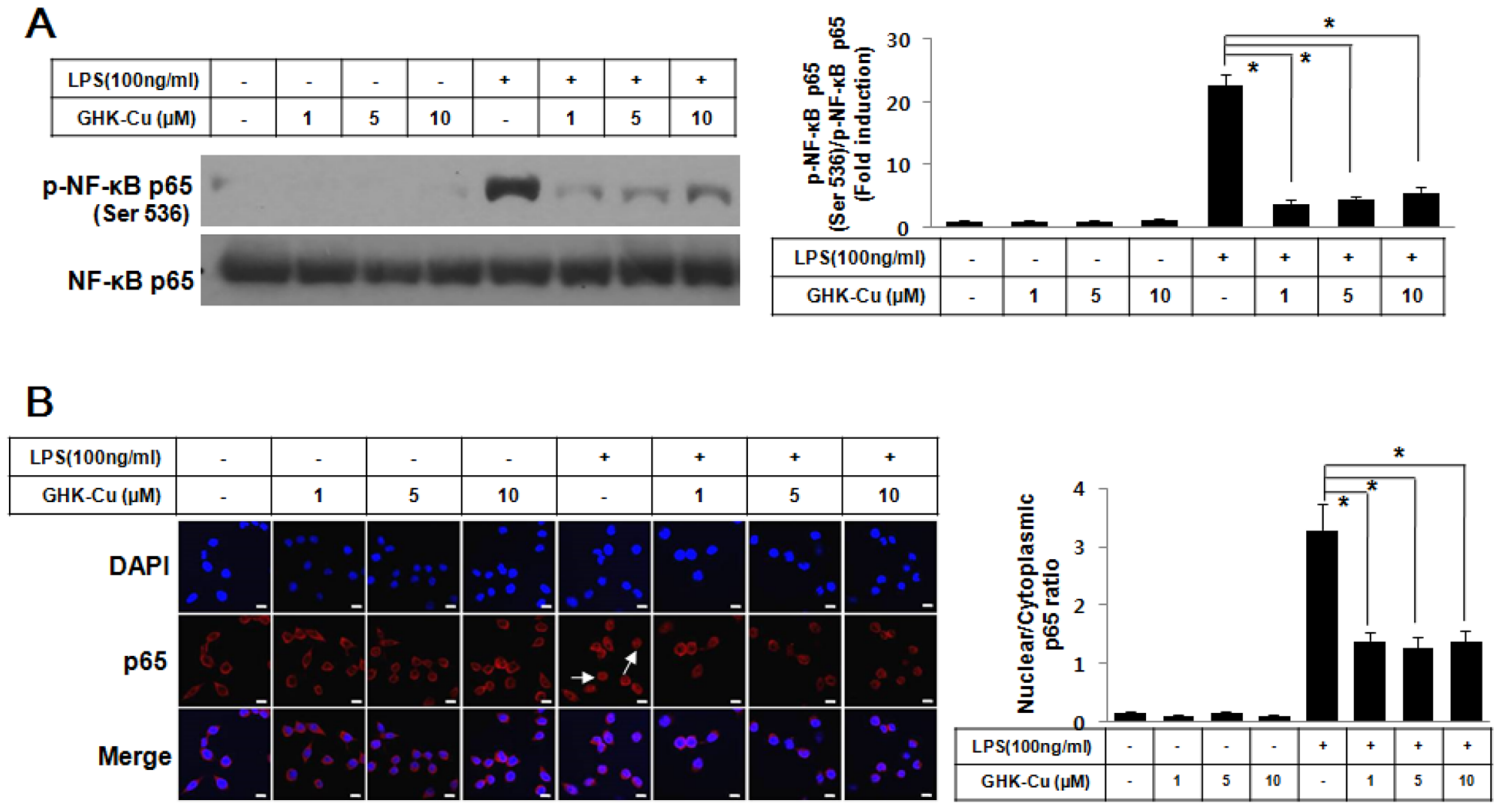
GHK-Cu blocked LPS-induced nuclear translocation of NF-κB p65 and phosphorylation of NF-κB p65 in LPS-induced RAW 264.7 cells activation **A.** Phosphorylation of p65 at Ser536 was assessed by western blotting of total cell lysates with a phosphorylation site-specific antibody. The values are the means ± SD after normalization based on total NF-κB p65 (n = 3). A representative blot is shown for each condition. The blots were subjected to a densitometric analysis and relative quantification. **B.** Immunofluorescence staining of NF-κB p65 and quantification of nuclear: cytoplasmic ratios of NF-κB p65 staining. A representative NF-κB p65 stain for these conditions is shown. White arrows indicate the localization of NF-κB p65 in the nucleus. The data represent the means ± SD from an analysis of three separate high-power-field images. *p < 0.05, statistically significant difference. CTL, control.

NF-κB plays an important role in the LPS-induced expression of pro-inflammatory proteins. To investigate the molecular mechanisms of the GHK-Cu-mediated inhibition of inflammatory mediators, we examined the effects of GHK-Cu on the activation of NF-κB. The transactivation potential of NF-κB is increased by the phosphorylation of the NF-kB p65 subunit [25]. We examined Ser536 phosphorylation of NF-κB p65 by western blotting. Figure 3A shows that LPS markedly induced the phosphorylation of NF-kB p65 at Ser536 in RAW 264.7 cells, while the treatment with GHK-Cu (1, 5, and 10 μM) inhibited the LPS-stimulated phosphorylation of NF-kB p65 at Ser536. In addition, the nuclear translocation of NF-κB was subjected to an analysis of immunofluorescence. LPS stimulation for 4 h caused the translocation of NF-κB p65 to the nucleus in RAW 264.7 cells (Figure 3B). However, GHK-Cu treatment effectively blocked the LPS-induced nuclear translocation of NF- κB p65 in LPS-treated RAW 264.7 cells (Figure 3B). These results suggested that GHK-Cu could suppress the LPS-induced activation of NF-κB signaling.

### GHK-Cu suppressed the activation of p38 MAPK signaling pathway in LPS-induced RAW 264.7 cells activation

**Figure 4:**
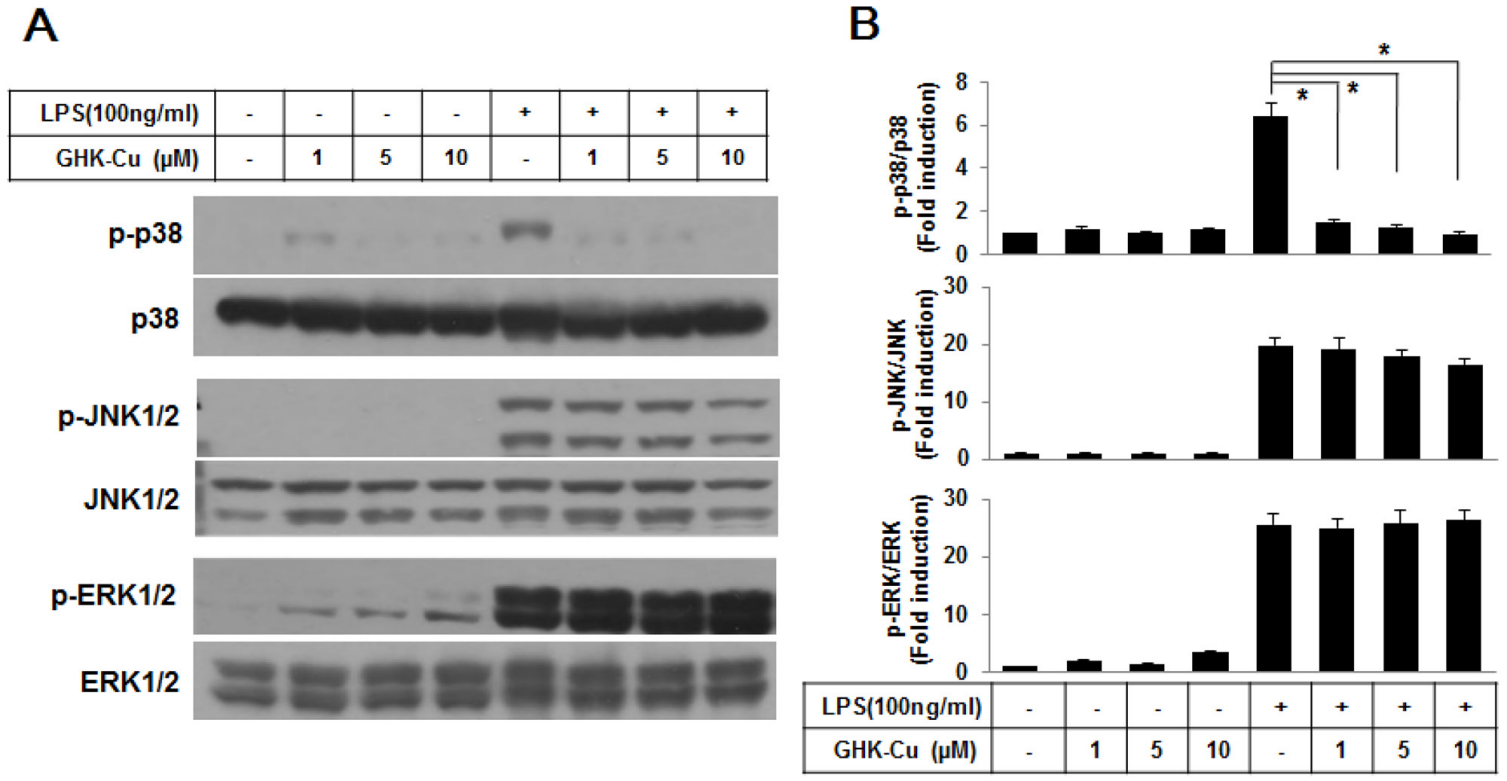
GHK-Cu suppressed the activation of the p38 MAPK signaling pathway in LPS-induced RAW 264.7 cells activation **A.** Western blotting was performed using a specific antibody for the detection of the phosphorylation of p38 MAPK, JNK1/2, and ERK1/2. p38 MAPK, JNK1/2, and ERK1/2 were used as loading controls. **B.** The blots were subjected to a densitometric analysis and relative quantification. The values are the means ± SD after normalization for p38 MAPK, JNK1/2, and ERK1/2 (n = 3). A representative blot is shown for each condition. *p < 0.05, statistically significant difference. CTL, control.

A number of studies have demonstrated that the MAPK pathway plays important roles in lung inflammatory injury. Therefore, we examined the effect of GHK-Cu on the LPS-stimulated phosphorylation of upstream kinases, including ERK1/2, JNK1/2, and p38 MAPK, in RAW 264.7 cells. As shown in Figure 4, GHK-Cu significantly inhibited the LPS-induced phosphorylation of p38 MAPK and slightly decreased phosphorylation of JNK1/2 whereas the phosphorylation of ERK1/2 was not affected. These results suggest that the anti-inflammatory effect of GHK-Cu might result from its modulation of the p38 MAPK signaling pathway.

### GHK-Cu attenuated lung damage in LPS-induced ALI in mice

**Figure 5:**
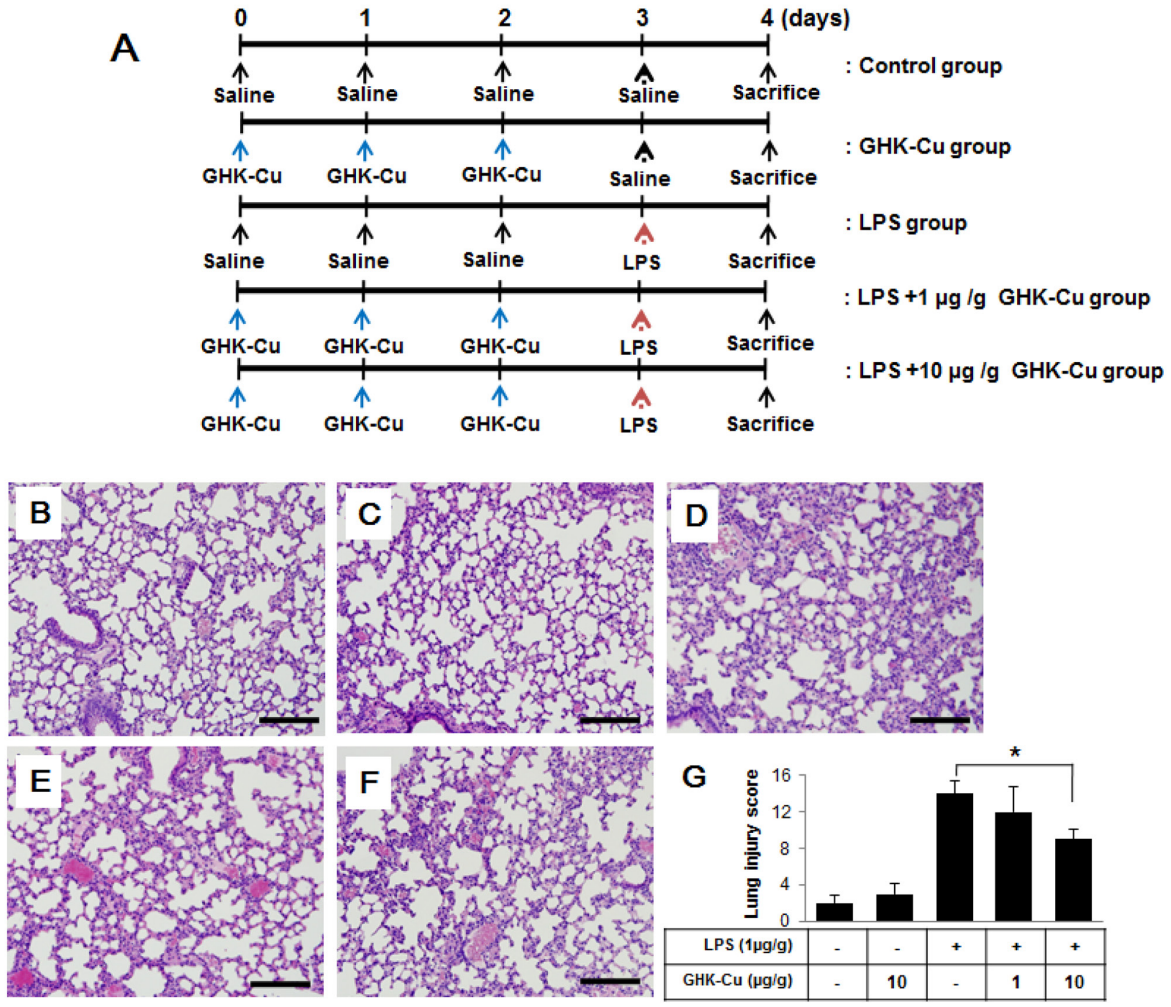
GHK-Cu attenuated LPS-induced acute pulmonary inflammation in mice Mice were injected i.p. with 1 or 10 μg/g GHK-Cu every 24 h for 3 days in 100 μl of saline, while control mice were injected i.p. with 100 μl of saline. Intratracheal LPS at 1 μg/g dissolved in 50 μl of saline or saline alone was administered 24 h after the third injection of GHK-Cu or saline. At 24 h after LPS administration, the mice were sacrificed and their left lungs were fixed. Then, tissue sections were stained with hematoxylin and eosin (H & E). **A.** Experimental scheme for LPS treatment with or without GHK-Cu. (n = 7 mice per group). **B.** CTL; **C.**10 μg/g GHK-Cu; **D.** LPS; **E.** LPS + 1 μg/g GHK-Cu; **F.** LPS + 10 μg/g GHK-Cu. **G.** Lung injury scores were calculated according to the sum of the levels of cell infiltration and damage levels as assessed from the lung sections. The data represent the means ± SD (n =7 per group). *p < 0.05, statistically significant difference. Scale bars represent 100 μm. CTL, control.

Mice were administered LPS with or without GHK-Cu pretreatment (Figure 5A). As shown in Figure 5B–5F, characteristic morphological changes were observed in lung sections after LPS administration, including notable immune cell infiltration, interstitial edema, alveolar wall thickness, and hemorrhage. GHK-Cu treatment did not affect histological changes and level of inflammatory cytokines compared to the control group (Figure 5B and 5C). Also, no toxicity was found after the GHK-Cu treatment. The morphological changes in the lung tissue of mice with ALI suggested that the ALI model was successfully triggered under the present experimental conditions. However, in the group that received pretreatment with GHK-Cu, the pathological changes in lung tissues were markedly mitigated, as evidenced by the significantly reduced lung injury score (Figure 5G). These results indicated that GHK-Cu had a protective effect on LPS-induced ALI.

### GHK-Cu increased antioxidant enzymes and decreased pro-inflammatory cytokines in LPS-induced ALI in mice

**Figure 6:**
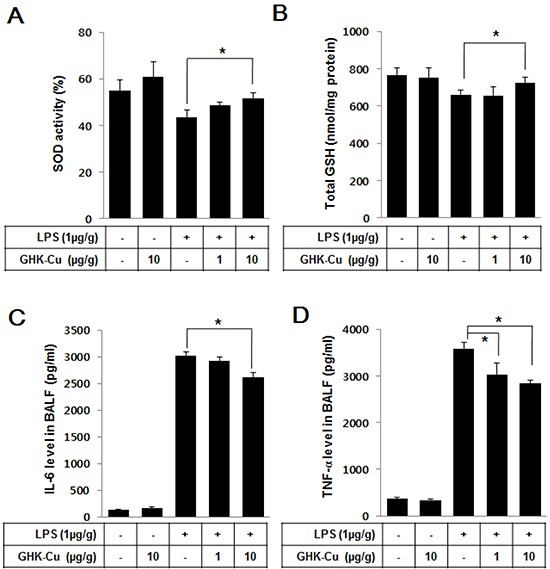
GHK-Cu increased antioxidant enzymes and decreased pro-inflammatory cytokines in LPS-induced ALI in mice At 24 h after LPS administration, the mice were sacrificed, and lung homogenates and BALF were prepared. **A.** Total intracellular SOD activity levels were detected using a colorimetric method. **B.** The level of glutathione (GSH) was measured in the lung homogenate. **C.** IL-6 and **D.** TNF-α were detected by ELISA. The data represent the mean ± SD (n = 3). *p < 0.05, statistically significant difference.

We investigated whether GHK-Cu can increase anti-oxidant enzymes and decrease pro-inflammatory cytokines in LPS-induced ALI in mice. As summarized in Figure 6A and 6B, only groups administered LPS exhibited decreased SOD activity and total GSH; however, the LPS + 10 μg/g GHK-Cu group exhibited significantly increased SOD activity and GSH compared to the LPS alone group. Pro-inflammatory cytokines in the BALF were measured by an ELISA at 24 h after LPS administration with or without GHK-Cu pretreatment. The concentrations of TNF-α and IL-6 cytokines in the BALF for the group administered only LPS were seen only with the highest individually. However, the response was markedly decreased by GHK-Cu in the LPS + 10 μg/g GHK-Cu group (Figure 6C and 6D). These results demonstrated that GHK-Cu increased antioxidant enzymes and reduced the expression of pro-inflammatory cytokines, which in turn alleviated lung damage caused by LPS-induced ALI.

### GHK-Cu reduced immune cell infiltration and alveolar permeability in the BALF

**Figure 7:**
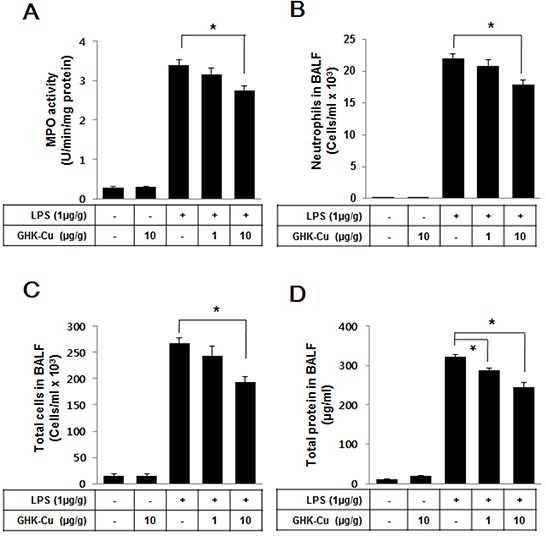
GHK-Cu reduced immune cell infiltration and total protein in BALF At 24 h after LPS administration, the mice were sacrificed and lung BALF was prepared. Cells in the BALF were collected and cytospin preparations were made. **A.** Myeloperoxidase (MPO) activity, **B.** neutrophils counts, **C.** total cell counts and **D.** total proteins were determined as described in Materials and Methods. The data represent the mean ± SD (n = 3). *p < 0.05, statistically significant difference.

Activated neutrophils have been linked to the production of oxidative stress, which is a critical factor in the pathophysiology of ALI. To further identify the anti-inflammatory properties of GHK-Cu, it is important to calculate immune cell infiltration into the lungs. Therefore, we evaluated alterations in immune cell infiltration in the lungs of LPS-administered mice with or without GHK-Cu pretreatment. MPO activity is a marker of neutrophils [26]. The level of lung MPO activity (Figure 7A) and infiltrated neutrophils (Figure 7B and Supplementary Figure S3) were markedly increased in the LPS group, whereas the LPS + 1 or 10 μg/g GHK-Cu group exhibited a trend toward lower levels in a dose-dependent manner. The total cell counts in the BALF were also lower in the LPS + 10 μg/g GHK-Cu group than the LPS group (Figure 7C). ALI exhibits intra-alveolar edema and increased alveolar permeability. We assessed lung permeability by measuring the total protein concentration in the BALF. The total proteins were significantly increased in the LPS-administered group, but decreased the LPS + 10 μg/g GHK-Cu group in the BALF (Figure 7D).

### GHK-Cu suppressed the phosphorylation of p38 MAPK, JNK1/2 and NF-κB p65 in LPS-induced ALI in mice

**Figure 8:**
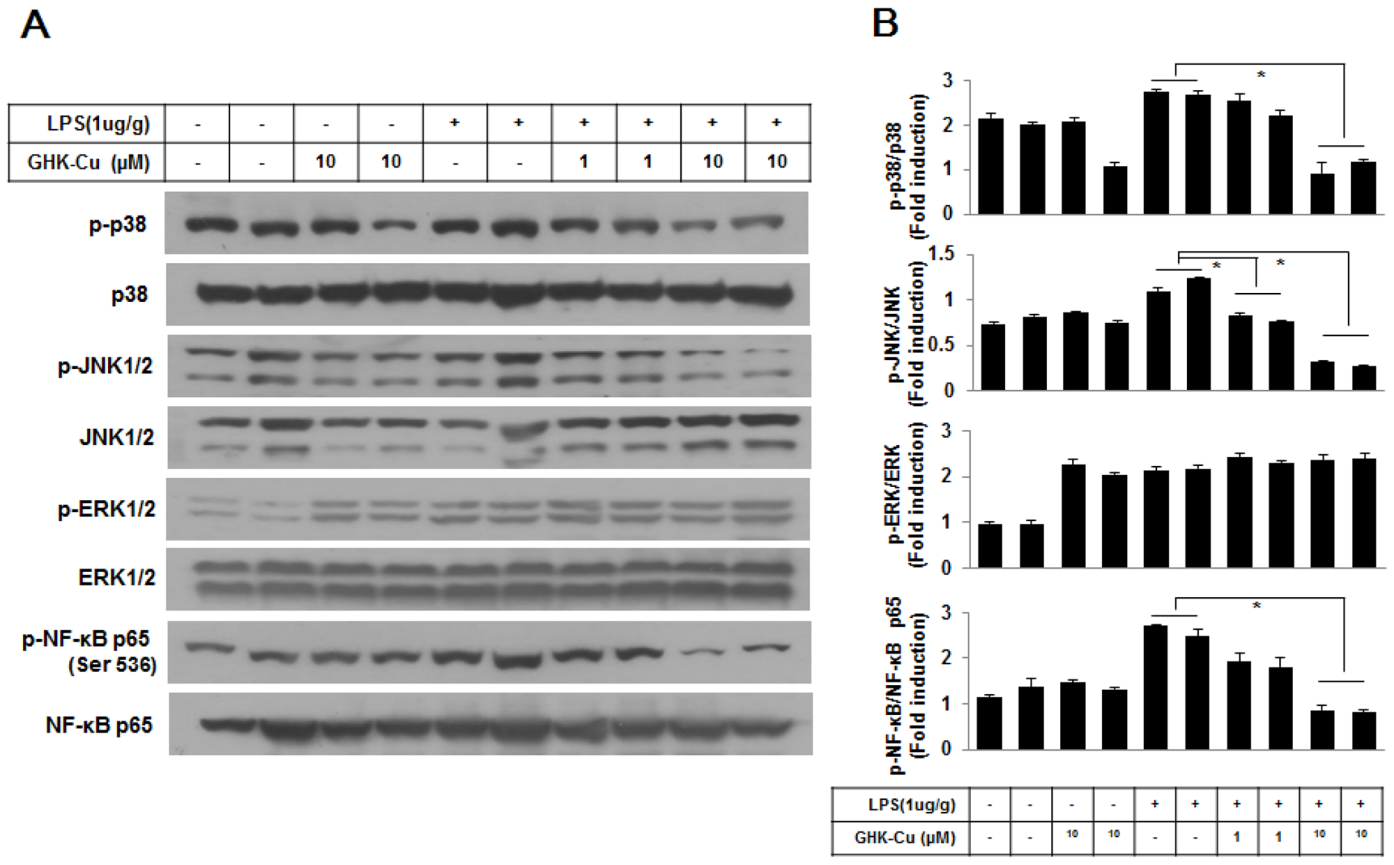
MAPKs and NF-κB signaling pathway is involved in the anti-inflammatory effect of GHK-Cu **A.** Phosphorylation of NF-κB p65 at Ser536, p38 MAPK, ERK1/2, and JNK1/2 was assessed by western blotting of lung homogenates with a phosphorylation site-specific antibody. NF-κB p65 and p38 MAPK, JNK1/2, and ERK1/2 were used as loading controls. **B.** The blots were subjected to a densitometric analysis and relative quantification. The values are the means ± SD after normalization for NF-κB p65, p38 MAPK, JNK1/2, and ERK1/2 (n = 3). A representative blot is shown for each condition. *p < 0.05, statistically significant difference.

The MAPK pathways have been demonstrated to participate in the activation of NF-kB in LPS-induced ALI. As shown in Figure 8, the results from Western blotting showed that GHK-Cu had little effect on LPS-induced phosphorylation of ERK1/2, however, it significantly reduced the phosphorylation of p38 MAPK and JNK1/2 in LPS + 10 μg/g GHK-Cu group compared to the LPS group. In parallel with phosphorylation of MAPK, the effect of GHK-Cu on phosphorylation of NF-κB p65 were measured by Western blotting. The phosphorylation of NF-κB p65 at Ser536 in lung increased significantly after LPS administration compared with control group, while the treatment with GHK-Cu reduced phosphorylation of NF-κB p65 at Ser536 in LPS + 10 μg/g GHK-Cu group compared to the LPS group (Figure 8).

## DISCUSSION

Copper peptides are naturally occurring small protein fragments that possess high affinity to copper ions in human serum and cerebrospinal fluid (PMID21898044). GHK-Cu accelerates wound healing, improves the success of transplanted skin, and has anti-inflammatory effects on the skin [19, 20]. It has a physiological role in anti-inflammatory responses and tissue remodeling. In this present study, we demonstrated that GHK-Cu exerts a protective effect in LPS-induced ALI.

Oxidative stress refers to elevated intracellular levels of ROS. High level of ROS, which that lead to cellular damage, are critical for LPS-induced inflammation [27]. Accordingly, we first performed experiments to determine the effects of GHK-Cu on intracellular ROS production. The pretreatment of cells with GHK-Cu significantly reduced LPS-induced ROS production. Few studies have examined the relationship between GHK-Cu and ROS [28]. Under normal physiological conditions, oxidative stress is improved by anti-oxidative enzymes acquired through SOD, catalase, and GSH. Notably, superoxide anions related to oxidative damage are converted to hydrogen peroxide by SOD, which is then metabolized to water via catalase or GSH [29]. In this study, we demonstrated that pretreatment with GHK-Cu increased SOD activity and total GSH in LPS-induced ALI. These observations suggest that GHK-Cu is capable of reducing serious lung damage by improving anti-oxidant enzymes.

ALI, an inflammatory disorder, is characterized, among other events, by the production of significant amounts of free radicals and nitrogen reactive species as well as inflammatory cytokines, such as TNF-α and IL-6. TNF-α is a pivotal proinflammatory cytokine and is regarded as an endogenous mediator. Excessive production of cytokines can be induced by inflammatory stimuli, such as LPS in macrophages, and it increases the immune response, which in turn results in inflammation [30]. Therefore, the inhibition of pro-inflammatory cytokines is a target for anti-inflammatory therapies. Our results showed that GHK-Cu could significantly suppress TNF-α and IL-6 expression *in vitro* and *in vivo*, suggesting that GHK-Cu is useful for the development of novel anti-inflammatory therapies.

The transcriptional regulator NF-κB plays a pivotal role in inflammation owing to its ability to induce the transcription of an array of inflammatory genes, especially those involved in the regulation of pro-inflammatory molecules [31]. Nuclear translocation of NF-κB p65 induced target gene activation. NF-κB p65 activation is associated with alveolar macrophage activity, which is the major source of inflammatory cytokines [32]. Our results demonstrate that GHK-Cu has the ability to inhibit the LPS-induced phosphorylation of NF-κB p65 at Ser536, an event associated with NF-κB activation, as well as the nuclear translocation of NF-κB p65 in macrophages. The therapeutic potential of inhibiting the NF-κB pathway in chronic inflammatory diseases and inflammatory bowel diseases has also been reported [33]. These findings concur with our finding that the transcriptional inhibition of proinflammatory mediators by GHK-Cu is associated with the blockade of the NF-κB signaling pathway.

In addition to NF-κB, LPS is a potent activator of the MAPK pathway, which regulates cytokine release in macrophages [34]. We explored whether the GHK-Cu has an effect on the activation of p38 MAPK, ERK1/2, and JNK1/2 signaling molecules in LPS-induced RAW 264.7 macrophages *in vitro* and ALI in mice *in vivo*. Our results showed that p38 MAPK, ERK1/2, and JNK1/2 activation occurred in LPS-induced ALI. However, GHK-Cu pretreatment markedly suppressed the LPS-induced phosphorylation of p38 MAPK, had little effect of JNK1/2, but not ERK1/2. Furthermore, we also examined the level of pro-inflammatory cytokines and phosphorylation of p38 MAPK and NF-kB to further investigate whether GHK-Cu can reduce anti-inflammatory response on the peritoneal macrophages. As expected, GHK-Cu decreased LPS-induced TNF-α and IL-6 secretion, and suppressed phosphorylation of p38 MAPK and NF-κB p65 (Supplementary Figure S2). N-acetylcysteine (NAC), a ROS scavenger, has been evaluated in various experimental conditions including ALI. Hsu et al., reported that NAC decreased p38 MAPK activity to the basal level as well as moderated LPS-induced inflammatory cytokine levels [35]. These results suggested that the suppression of p38 MAPK phosphorylation by GHK-Cu is involved in the inhibition of the LPS-induced production of proinflammatory mediators in LPS-induced ALI.

ALI is characterized by excessive neutrophil infiltration into the lung, and neutrophils serve as a therapeutic target to attenuate inflammation in ALI. Although neutrophil activation is vital for host defense, excessive activation leads to tissue damage by cytotoxic and immune cell-activating agents, such as proteinases, cytokines, and ROS [36]. Therefore, to determine whether GHK-Cu affects neutrophil infiltration to alleviate ALI, we analyzed neutrophil infiltration into the lung by detecting total MPO, an enzyme released from activated neutrophils, and the neutrophil count in lung BALF. These results suggested that GHK-Cu can mitigate neutrophil infiltration into the lung during LPS-induced ALI.

**Figure 9:**
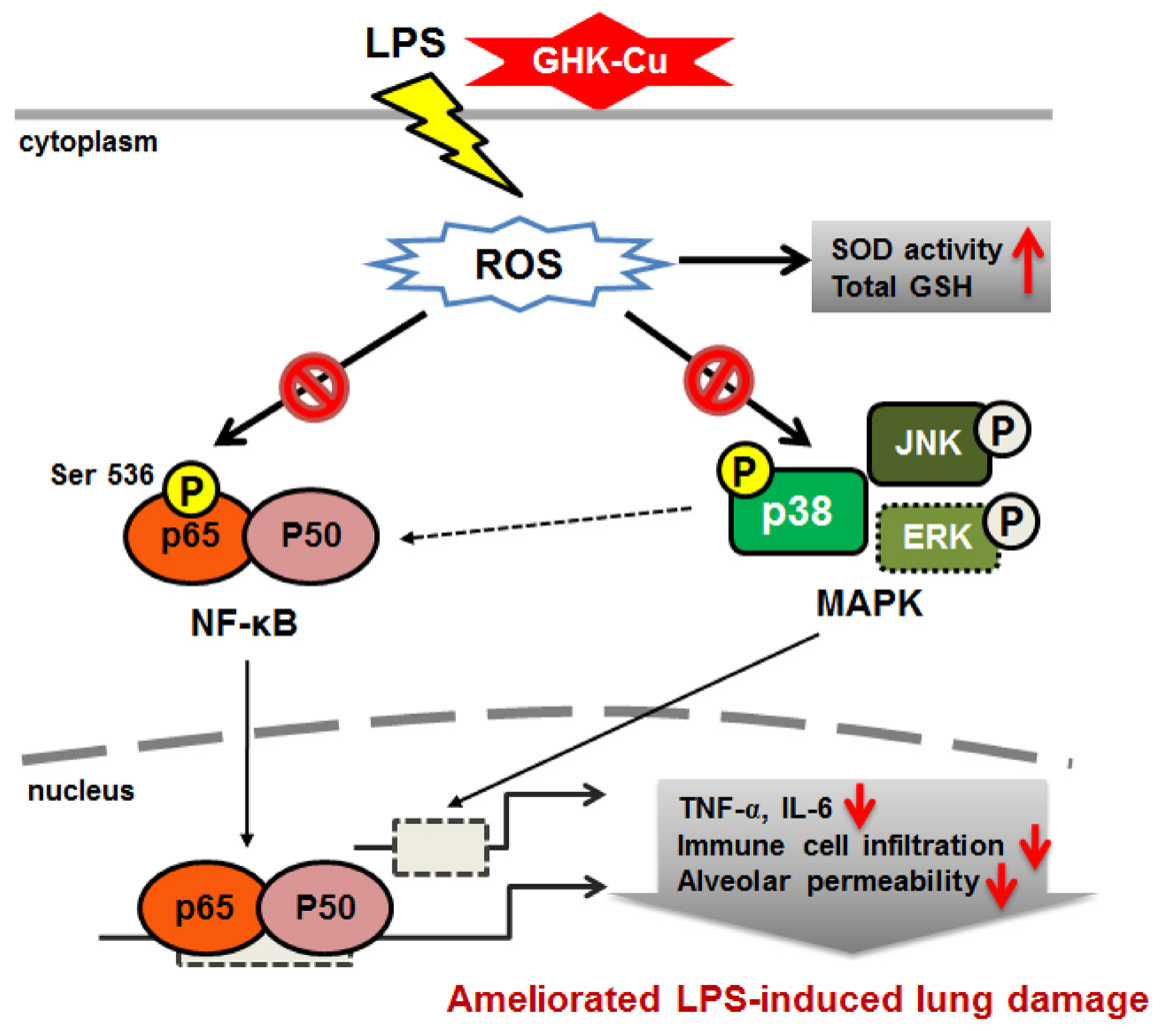
Schematic diagram showing the proposed mechanisms underlying the attenuation of LPS-induced inflammatory response by GHK-Cu

To the best of our knowledge, this is the first study to investigate the protective effect of GHK-Cu via its anti-inflammatory and anti-oxidant properties and its possible mechanisms in LPS-induced ALI (Figure 9). Our results provide valuable insight into the molecular actions of GHK-Cu and revealed its underlying mechanisms. These findings indicated that GHK-Cu are a promising agent for the clinical prevention and therapy of inflammatory disease.

## MATERIALS AND METHODS

### Materials

GHK-Cu (MW 340.38; soluble in water) was provided by Bioceltran Corporation Ltd., (Chuncheon, Korea). Lipopolysaccharide (*Escherichia coli* O111:B4), thioclycollate and 2′,7′-dichlorofluorescin-diacetate (DCFDA) were purchased from Sigma (MO, USA). Antibodies against phospho-ERK1/2 (#4370), total ERK1/2 (#4695), phospho-p38 MAPK (#4511), total p38 MAPK (#9212), phospho-JNK1/2 (#4668), total JNK1/2 (#9252), phospho-NF-κB p65 (Ser536, #3033) and β-actin (#3700) were purchased from Cell Signaling (MA, USA). Antibodies against NF-κB p65 (sc-372) was purchased from Santa Cruz Biotechnology (CA, USA).

### Cell cultures, preparation of peritoneal macrophages, cell proliferation and cell stimulation

The murine macrophage cell line RAW 264.7 cells were purchased from Korea Cell Line Bank (KCLB, Seoul, Korea). Peritoneal macrophages were harvested 4 days after the i.p. injection of 3% thioglycollate. Macrophages were washed and plated in 24-well plates at 0.5×10^6^ cells per well. After incubation for 2 h at 37°C, the wells were washed 3 times to remove all nonadherent cells. Finally, the culture medium was replaced. Cells were grown in RPMI 1640 medium containing 10 % fetal bovine serum (Hyclone, USA) in a 37°C humidified incubator containing 5 % CO_2_ and 95 % air. Cells were pretreated without or with GHK-Cu at various concentrations for 18 h. The cells were then stimulated with LPS (100 ng/ml) for indicated time period. The culture supernatants or cells were collected for the following analysis. Cell proliferation was assessed using 3-(4,5-dimethylthiazol-2-yl)-2,5-diphenyltetra-zolium bromide (MTT; Sigma-Aldrich) assay.

### Measurement of intracellular ROS (DCFDA)

The level of intracellular ROS was detected using cell permeable fluorescent probe 2′, 7′-dichlorofluorescin-diacetate (DCFDA). Intracellular detection of ROS was achieved by incubating cells with 10 μM CM-DCFDA for 30 min at 37°C prior to LPS and GHK-Cu treatment. Cells were then detached by gentle scraping with a cell scraper and analyzed by Accuri-C6 flow cytometry using FL1 channel (BD biosciences, USA).

### Immunostaining

Cells were seeded on round glass cover slips in 24-well plates 24 h prior to treatment with GHK-Cu and LPS. After LPS treatment, the samples were washed with cold PBS and fixed in 4 % paraformaldehyde in PBS for 15 min at room temperature. The cells were then permeabilized with 0.2 % Triton X-100 in PBS for 10 min at room temperature and incubated overnight at 4°C with either anti- NF-κB p65 antibody. After 3 washes with PBS, the cells were incubated with Alexa Fluor 546-conjugated goat-anti rabbit IgG (Invitrogen, CA, USA) for 1 h at room temperature and then washed again with PBS. The images were captured using a confocal laser-scanning microscope.

### Western blot analysis

Whole cell lysates and lung homogenates were prepared with RIPA lysis buffer containing with a protease inhibitor cocktail (GenDEPOT; P3100, P3200). The protein concentrations in the lysates were determined by Bicinchoninic (BCA) Protein Assay Kit (Thermo Scientific, IL, USA: 23225), following the manufacturer's instructions. Then, the 20 ug of isolated soluble proteins was mixed with 5X SDS-PAGE sample buffer (Tech&Innovation, Chuncheon, Korea; BSS-9005), electrophoresed in a 10 % polyacrylamide gel and transferred to nitrocellulose transfer membrane 0.45 mm (BIO-RAD, CA, USA; 162-0115). The membranes were blocked with 5 % skim milk (dilution in TBS with 0.05 % Tween 20 (TBST)) and incubated with primary anti-bodies (1:1000, dilution in TBST) for overnight at 4°C. After sufficiently washed with TBST, the membranes were incubated with polyclonal anti-rabbit/mouse HRP-conjugated secondary antibodies for 1 h at room temperature. After that, the membranes were washed with TBST and detected using an ECL detection solution.

### Animals

Thirty five male C57BL/6 mice, 10 weeks old, were purchased form Doo Yeol Biotech (Seoul, Korea). Animals were housed in the Animal Facility of Kangwon National University with an environmentally controlled room (23°C ± 2, 55 % ± 5 % relative humidity) with 12 h dark-light cycles. During the experiment, the animals were fed with a standard laboratory chow and water. All experiments were approved and followed the regulations of the Institutional Animal Care and Use Committee (IACUC, KW-130503-1, Kangwon National University, Korea).

### Model of LPS-induced acute lung injury

ALI was induced by intratracheal administration of 1 μg/g LPS in 50 μl of saline. With this model, ALI, as characterized by neutrophils infiltration into the lung interstitium and airways, development of interstitial edema, and increased pulmonary proinflammatory cytokine production, occurs after injection of LPS, with the greatest degree of injury being present 24 h after LPS exposure [37]. Briefly, mice were anesthetized with Zoletil 50 (Virbac, France; 30mg/kg, i.p.). The tongue was then gently extended, and the LPS solution was deposited into the pharynx [38]. Thirty five male C57BL/6 mice were randomly divided into 5 groups (n=5): control group, LPS group, GHK-Cu group (10 μg /g), low dosage group (1 μg/g GHK-Cu + LPS), and high dosage group (10 μg /g GHK-Cu + LPS). For the GHK-Cu pretreatment groups, mice were injected intraperitoneally with GHK-Cu (1 or 10 μg/g, every 24 h, for 3 days) dissolved in saline before intratracheal administration of LPS. The mice in the control group were administrated with saline instead. 24 h later, mice were sacrificed. There were no deaths associated with this model for GHK-Cu and/or LPS administration.

### Histopathological analysis

To examine the histological examination, the left lung lobes were trimmed and fixed by immersion in 10 % neutral formalin for 24 h. Paraffin-embedded lungs were cut into 4 μm thick sections and subsequently stained with hematoxylin and eosin for histological analysis. Lung injury was graded from 0 (normal) to 4 (severe) in four categories. Lung injury score was evaluated according to the sum of the damage level such as the neutrophil infiltration, congestion, edema and alveolar wall thickness. [39].

### Harvest of lungs and bronchoalveolar lavage fluid (BALF)

Lungs were harvested 24 h after LPS administration. BALF was collected from mice to analyze the cell counts, total protein content and cytokine production. Mice were sacrificed, and their tracheas were immediately lavaged two times via a catheter with 1 ml of ice-cold PBS. BALF collected was centrifuged at 2,000 rpm for 5 min at 4°C. The supernatants were collected and then stored at −80°C until the cytokine assay and determination of total protein content. Differential cell counts were assessed on cytological preparations obtained by cytospin centrifuge (Cellspin, Hanil, Korea) of 100 μl of the diluted BALF (1 × 10^6^ cells/mL in ice-cold PBS). The slides were fixed and stained with Hema-3 stain (Thermo Fisher Scientific) according to the manufacturer's instructions, and cells were counted under a light microscope. At least 400 cells were counted for each preparation. The number of neutrophils were expressed as the absolute number from the total cell counts.

### Measurement of Myeloperoxidase (MPO) activity and nitrite determination

MPO activity was measured using a minor modification of a previously described method [38]. In brief, lung tissue was homogenized in 0.5 ml of 0.5 % hexadecyltrimethyl ammonium bromide in 50 mM potassium phosphate buffer (pH 6.0). The homogenate was centrifuged at 12,000 rpm for 15 min at 4°C and the supernatant was collected for assay MPO activity as determined by measuring the H_2_O_2_-dependent oxidation of *o*-dianisidine solution (3,3′-dimethoxybenzidine dihydrochloride in potassium phosphate buffer, pH 6.0) at 450 nm using an Epoch microplate spectrophotometer (BioTek, VT, USA). NO detection in culture media was performed using Griess reagent (Sigma–Aldrich, Saint Louis, MO) at 540 nm.

### Determination of SOD and GSH activity

SOD activity was assessed using the commercial SOD determination kit (Sigma–Aldrich, Saint Louis, MO). The rate of the activity of SOD was measured the absorption spectrum about formazan form of WST-1 (2-(4-lodophenyl)-3-(4-nitrophenyl)-5-(2,4-disulfophenyl)-2H-tetrazolum, monosodium salt), highly water-soluble tetrazolium salt, that generate a water-soluble formazan dye with reduction of a superoxide anion. Consequentially, amount of superoxide anion is proportional to the absorbance at 440 nm, and means inhibition activity of SOD. The assay for quantitative determination of glutathione (GSH) levels was conducted as previously described method by Rahman et al. [40]. For the GSH assay, the lungs were harvested, washed in PBS, and then homogenated in 0.1 M phosphate buffer containing 5 mM EDTA, 0.1 % (vol/vol) Triton X-100, and 0.6 % (wt/vol) sulfosalicylic acid. The cellular debris was collected by centrifugation, the supernatant concentration were determined by BCA assay Kit. The proteins were incubated with 0.67 mg/ml DTNB and 10 U/ml glutathione reductase in 0.1 M potassium phosphate buffer with 5 mM EDTA disodium salt, pH 7.5. After 30 s, 0.67 mg/ml b-NADPH was added. The actual total GSH concentration in the samples were determined by calculated linear regression values from standard curve every 30 s for a total time of 2 min at 412 nm absorbance.

### ELISA for TNF-α and IL-6 cytokines

RAW 264.7 cells (5 × 10^5^ per well) were seeded in 24-well plates containing RPMI 1640 supplemented with 10 % FBS for 1 day to become nearly confluent. On the second day, the cells were pretreated with the indicated concentrations of GHK-Cu 18 h before treatment with LPS at 37°C for 4 h. The cultured media were collected and stored at - 80°C for the following cytokine assay. The bronchoalveolar lavage fluid (BALF) supernatant was collected after centrifugation and stored at - 80°C before the cytokine assay. Immunoreactive TNF-α and IL-6 were measured by Duoset enzyme-linked immunosorbent assay (ELISA) development kits (R&D systems, MN, USA) according to the manufacturer's instruction.

### Statistical analysis

All the statistical data were analyzed by GraphPad Prism 5.0 (GraphPad Software, CA, USA). The results were expressed as the mean ± SD. Comparisons between two groups were performed using an unpaired student's *t*-test. One-way analysis of variance (ANOVA) was used for comparisons between multiple groups. A value of *p* < 0.05 was considered statistically significant.

## SUPPLEMENTARY FIGURES


