# Supplementary material for: The tri-peptide GHK-Cu complex ameliorates lipopolysaccharide-induced acute lung injury in mice
Source: Oncotarget. 2016 Aug 10;7(36):58405–17. doi: 10.18632/oncotarget.11168 (PMC5295439; doi:10.18632/oncotarget.11168)
Supplement: Supplementary file 1 [file oncotarget-07-58405-s001.pdf]

The tri-peptide GHK-Cu complex ameliorates lipopolysaccharide-induced acute lung injury in mice

SUPPLEMENTARY FIGURES

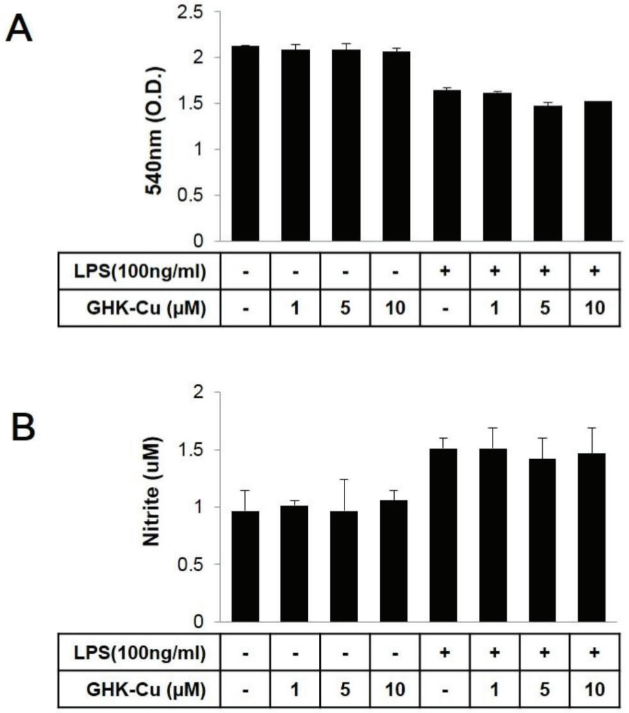

Supplementary Figure S1: Effect of GHK-Cu on cell proliferation and NO production in RAW 264.7 cells. A. MTT assay of RAW 264.7 cells after treatment with LPS with or without various concentration of GHK-Cu. **B.** The nitrite levels in the culture supernatants were assessed by a modified Griess assay. The data represent the mean ± SD (n = 3).

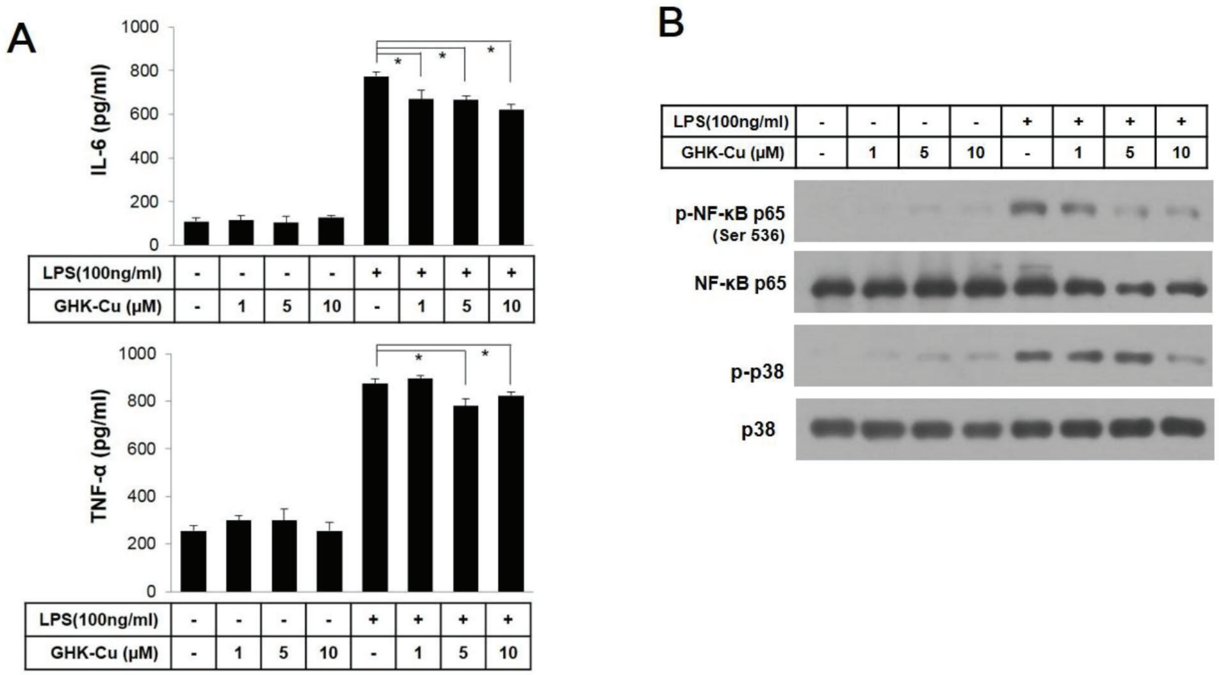

Supplementary Figure S2: GHK-Cu reduced pro-inflammatory cytokines and suppressed phosphorylation of NF-κB p65 at Ser536 and p38 MAPK in LPS-induced peritoneal macrophages. A. IL-6 and TNF-α were detected by ELISA. The data represent the mean ± SD (n = 3). *p < 0.05, statistically significant difference. **B.** Western blotting was performed using a specific antibody for the detection of p38 MAPK and NF-κB p65 phosphorylation. NF-κB p65 and p38 MAPK were used as loading controls.

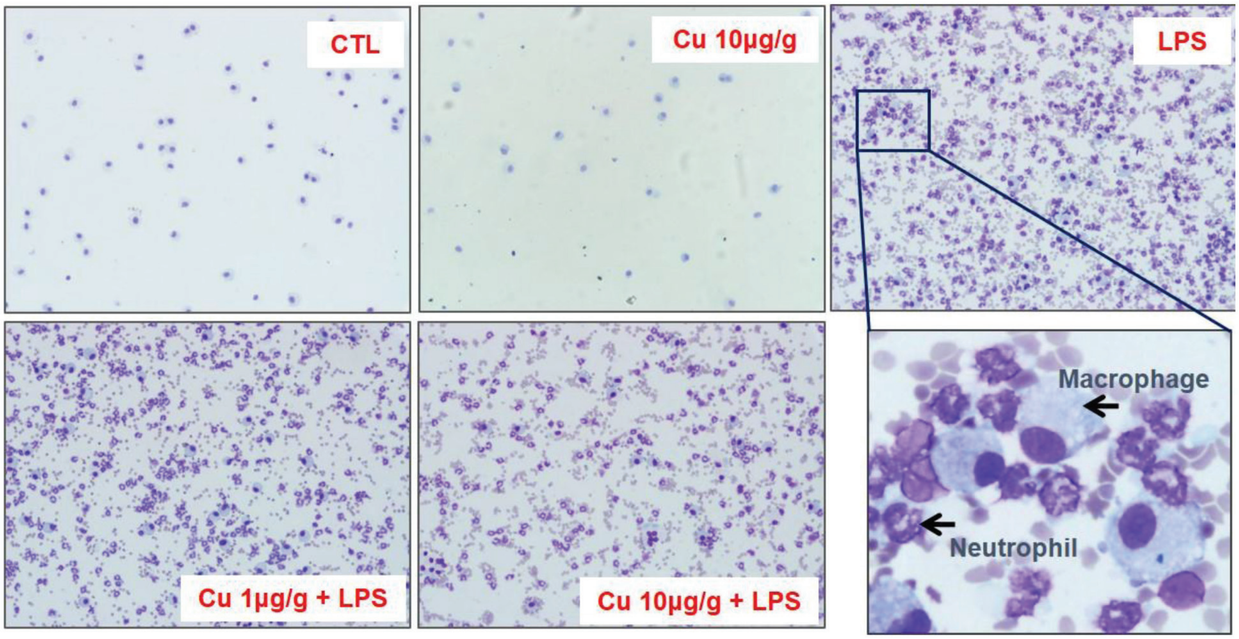

Supplementary Figure S3: GHK-Cu reduced immune cell infiltration. Representative cytospin images showing immune cells in the BALF. The infiltrated neutrophils were markedly increased in the LPS group, whereas the LPS + 1 or 10 μg/g GHK-Cu group exhibited a trend toward lower levels in a dose-dependent manner. Original magnification ×200.
